# Choice of antiretroviral drugs for continued treatment scale-up in a public health approach: what more do we need to know?

**DOI:** 10.7448/IAS.19.1.20504

**Published:** 2016-02-02

**Authors:** Marco Vitoria, Andrew M Hill, Nathan P Ford, Meg Doherty, Saye H Khoo, Anton L Pozniak

**Affiliations:** 1 Department of HIV/AIDS, World Health Organization, Geneva, Switzerland; 2 Department of Pharmacology and Therapeutics, Liverpool University, Liverpool, UK; 3 St Stephens AIDS Trust, Chelsea and Westminster Hospital, London, UK

**Keywords:** antiretroviral treatment, Universal Access, nucleoside analogues, integrase inhibitors, protease inhibitors, non-nucleosides, pregnancy, tuberculosis

## Abstract

**Introduction:**

There have been several important developments in antiretroviral treatment in the past two years. Randomized clinical trials have been conducted to evaluate a lower dose of efavirenz (400 mg once daily). Integrase inhibitors such as dolutegravir have been approved for first-line treatment. A new formulation of tenofovir (alafenamide) has been developed and has shown equivalent efficacy to tenofovir in randomized trials. Two-drug combination treatments have been evaluated in treatment-naïve and -experienced patients. The novel pharmacokinetic booster cobicistat has been compared to ritonavir in terms of pharmacokinetics, efficacy and safety. The objective of this commentary is to assess recent developments in antiretroviral drug treatment to determine whether new treatments should be included in new international guidelines.

**Discussion:**

The use of first-line treatment with tenofovir and efavirenz at the standard 600 mg once-daily dose should remain the first-choice standard of care treatment. Evidence supporting a switch to efavirenz 400 mg once daily or integrase inhibitors is sufficient to consider these drugs as alternative first-line options, but more data are needed on their use in pregnant women and people with TB co-infection. The use of new formulations of tenofovir is currently too preliminary to justify immediate adoption and scale-up across HIV programmes in low- and middle-income countries. The evidence supporting use of two-drug combinations is not considered strong enough to justify changed recommendations from use of standard triple drug combinations. Cobicistat does not offer significant safety advantages over ritonavir as a pharmacokinetic booster.

**Conclusions:**

For continued scale-up of antiretroviral treatment in low- and middle-income countries, use of first-line triple combinations including efavirenz 600 mg once daily is supported by the largest evidence base. Additional studies are underway to evaluate new treatments in key populations, and these results may justify changes to these recommendations.

## Introduction

In 2013 the World Health Organization (WHO) consolidated guidelines for the diagnosis, treatment and care of people with HIV [1]. These guidelines recommended a preferred first-line treatment with a combination of tenofovir disoproxil fumarate (TDF), lamivudine (3TC) or emtricitabine (FTC) and efavirenz (EFV), with second-line treatment using two nucleoside analogues and a boosted protease inhibitor (PI) [1]. Given the results from the START and TEMPRANO studies [2,3], the WHO has also issued early-release guidelines in 2015 to recommend immediate initiation of antiretroviral treatment for all HIV-positive people, regardless of CD4 count [4].

There are currently 37 million people infected with HIV worldwide and 15 million taking antiretrovirals [5]. In 2014, UNAIDS set the “90–90–90” target, aiming to diagnose 90% of all HIV-positive people, provide antiretroviral therapy for 90% of those diagnosed and achieve undetectable HIV RNA for 90% of those treated, by the year 2020 [6]. If there were no new HIV infections in the next five years, meeting these new targets would involve 33 million people being diagnosed, 30 million taking antiretrovirals and 27 million with HIV RNA suppression. However, meeting the UNAIDS targets by 2020 would also need to include testing and treating all those newly infected in the next five years. There were two million new infections in 2014 alone, so it is likely that at least 35 million people would need to be on treatment by 2020 to include newly infected people in the 90–90–90 targets.

Since the WHO treatment guidelines were released in 2013, there have been five key developments in antiretroviral drug treatment:

1. A lower dose of EFV – 400 mg once daily – has shown non-inferior efficacy and lower risks of EFV-related adverse events compared to the standard 600 mg once-daily dose [7].
2. Integrase inhibitors, including dolutegravir (DTG), elvitegravir (ELV) and raltegravir (RAL), have been introduced for first, second and subsequent lines of treatment in North America and Europe. In the SINGLE trial, first-line use of dolutegravir showed non-inferior virological suppression rates but lower risks of adverse events, compared with EFV 600 mg once daily [8].
3. A new prodrug formulation of tenofovir, alafenamide fumarate (TAF), has been evaluated in clinical trials, compared with the original prodrug form of TDF [9].
4. A new pharmacokinetic booster – cobicistat – has been co-formulated with ELV and some PIs and compared with the original booster drug ritonavir [10].
5. Two-drug combinations of a boosted PI with either an integrase inhibitor or a nucleoside analogue have been compared with standard triple-drug combinations, both in treatment-naïve patients and as a switching option for people with HIV RNA suppression [11,12].

Increasing the number of people on antiretroviral treatment from 15 million to 35 million will require careful choices of drugs. The fixed dose combination of TDF + 3TC (or FTC) +EFV 600 mg once daily is currently being used by the majority of people treated worldwide, and its use is likely to grow [13]. There has been a gradual shift of patients from older combinations (e.g. those including stavudine, didanosine, zidovudine or nevirapine) onto this single-tablet regimen. This shift is mainly justified by the better tolerability and convenience of this combination compared to the commonly used alternatives at the time. Cost is also a key driver of treatment uptake.

Table 1 shows the potential prices for new treatment combinations, based on recent forecasts [13,14]. If 35 million people were to be treated, small savings in the unit cost of antiretrovirals could translate into hundreds of millions of dollars saved per year, which could be used for additional treatment and care.

**Table 1:** Target prices for key first-line combination treatments in low or low-middle income countries

Combination treatment	Estimated price per patient-year	Reference
TDF/3TC/ATV/r	$279	13
TDF/FTC/ELV/COBI	$184	14
ABC/3TC/DTG	$179	14
TDF/FTC/EFV600	$144	13
TDF/3TC/EFV600	$130	13
TDF/3TC/EFV400	$100 to $110	13
TAF/3TC/DTG	$60	14
DTG/3TC	$46	14

3TC, lamivudine; ABC, abacavir; ATV/r, atazanavir/ritonavir; COBI, cobicistat; DTG, dolutegravir; EFV400, efavirenz 400 mg; EFV600, efavirenz 600 mg; ELV, elvitegravir; FTC, emtricitabine; TAF, tenofovir alafenamide fumarate; TDF, tenofovir disoproxil fumarate.

The complexity and cost of first-line PI-based treatment remains significantly higher than the use of EFV or nevirapine. Moreover, elvitegravir/cobicistat and RAL are predicted to cost significantly more than dolutegravir to produce. Use of 3TC is cheaper than FTC, and the equivalent efficacy of these two nucleoside analogues has been established from a systematic review [15], although this should be updated if more randomized head-to-head trials comparing these drugs are completed. There could be substantial cost savings from a switch to either EFV 400 mg once daily, DTG or TAF. In the longer term, the use of two-drug combinations could save additional costs.

In order to be included in WHO global treatment guidelines, new treatments or strategies typically will have been evaluated in large, well-powered randomized trials for at least two years. In addition, the treatments need to be evaluated in key populations who may not normally be recruited into Phase 3 trials, such as pregnant women and people co-infected with TB. Drug interactions with treatments commonly used in low-income countries, such as rifampicin, would need to be well understood.

The purpose of this commentary is to summarize and discuss considerations for incorporating these new HIV treatments into future updates of global treatment guidelines and to identify what additional research is required.

## Discussion

### EFV 400 mg once daily

The ENCORE-1 trial has shown non-inferior efficacy of EFV at low dose 400 mg versus the standard dose of 600 mg, both given in combination with TDF/FTC [7]. Results at 96 weeks also showed no difference in the risk of treatment-emergent resistance between the arms and a lower number of EFV-related adverse events for the EFV 400 mg dose. Summary results are shown in Table 2. The ENCORE 1 trial investigators concluded that 400 mg EFV should be recommended as part of routine care (although caution was noted with rifampicin co-administration). The efficacy results in the ENCORE-1 were consistent across different races and CYP2B6 polymorphisms, which are known to affect EFV concentrations [16]. In a pharmacokinetic sub-study, patients receiving the 400 mg once-daily dose had EFV Cmin levels 27% lower than those receiving the 600 mg dose, but this did not correlate with lower efficacy. In the original dose-ranging study of EFV, there was no difference in efficacy between EFV doses of 200, 400 and 600 mg daily, given first line in combination with ZDV + 3TC [17].

**Table 2:** Summary Week 96 efficacy and safety results from randomized trials of new treatments versus EFV 600 mg once daily [7,8]

Clinical trial	New third drug	EFV 600 mg
ENCORE-1		
Treatment arms	TDF/FTC/EFV 400 mg	TDF/FTC/EFV 600 mg
Sample size	312	309
HIV RNA <50 copies/mL	86.3%	86.7%
Virological failure	*n=*10	*n=*13
Drug resistance	*n=*2	*n=*3
EFV-related adverse events	37.7%	47.9%
Discontinuation for EFV-related adverse events	8.3%	15.5%
Single		
Treatment arms	ABC/3TC/DTG	TDF/FTC/EFV 600 mg
Sample size	414	419
HIV RNA <50 copies/mL	80%	72%
Virological failure	6%	6%
Drug resistance	0%	1.4%
Grade 2 to 4 clinical adverse events	14%	28%

3TC, lamivudine; ABC, abacavir; DTG, dolutegravir; EFV, efavirenz; FTC, emtricitabine; TDF, tenofovir disoproxil fumarate.

There is a large body of evidence to support the use of EFV 600 mg in a range of settings, leading to an estimated 15 million person-years of experience using EFV 600 mg in combination with TDF and 3TC (or FTC) [1]. This evidence provides a level of confidence that does not currently exist with the 400 mg dose, including the effectiveness of this dose in patients receiving concomitant rifampicin-based treatment for tuberculosis and the efficacy of this dose during pregnancy. Thus, the transition from the 600 mg to the 400 mg dose of EFV needs to be carefully managed, following ongoing review of clinical trials of the 400 mg dose in pregnancy and rifampicin-based treatment.

Pharmacokinetic studies showed that rifampicin-based treatment leads to short-term reductions in EFV drug levels during the first one to two weeks of treatment, but after longer-term treatment in combination with rifampicin-based combinations increases in EFV drug levels have been observed consistently across several studies [18]. However these overall trends could differ by ethnicity, as suggested in the STRIDES study [19]. The efficacy of EFV-based treatment is similar for people either taking or not taking rifampicin-based treatment (in contrast to nevirapine, which shows lower efficacy when co-administered with rifampicin) [20]. The alternative non-nucleoside reverse transcriptase inhibitor (NNRTI) rilpivirine is contraindicated for use with rifampicin because of drug interactions [21]. It is not clear whether the same consistent efficacy will be seen for the lower, 400 mg dose of EFV.

With respect to pregnancy, a recent review of six studies concluded that there was a limited effect on the pharmacokinetics of EFV at the standard 600 mg once-daily dose during the third trimester of pregnancy [22]. Rates of vertical transmission of HIV in these studies were low. Two more recent studies suggested that pregnancy may lower EFV plasma concentrations [23,24]. However, if EFV 400 mg is known to be effective from the ENCORE-1 study [7], the 600 mg dose of EFV is likely to retain its efficacy in pregnancy despite reductions in plasma concentration of this size.

To help address these questions, a new pharmacokinetic study of EFV 400 mg for pregnant women is currently recruiting, and a pharmacokinetic study of EFV 400 mg for use with rifampicin is in the planning phase [25].

Overall, even if the EFV 400 mg dose could not be universally adopted in all populations, it could be a treatment option to be considered for countries that are able to be flexible on dosing: the majority of patients could benefit from the improved safety profile and lower costs of the 400 mg dose, whereas the 600 mg dose could be used for pregnant women and those taking rifampicin-based treatments. It should be noted that the combination of TDF, 3TC and EFV will be fully generic in most countries worldwide by 2017, and so there is the potential for prices to fall close to production costs worldwide, if imports of cheap generic drugs can be properly organized. By contrast, the patents on the use of DTG and TAF extend for at least another 10 years [26], so costs of these drugs could be significantly higher than the TDF/3TC/EFV combination in many middle-income countries that do not qualify for minimum prices, such as in Eastern Europe, South America and parts of South-East Asia.

### Dolutegravir 50 mg once daily

In the SINGLE study, treatment-naïve patients were randomized to receive either abacavir (ABC)/3TC/DTG or TDF/FTC/EFV for 96 weeks [8]. The results showed an improved safety profile for DTG once daily compared with EFV 600 mg once daily, but no improvement in the risk of virological endpoints. The risk of treatment-emergent drug resistance after 96 weeks was 1.4% in the EFV arm versus 0% in the dolutegravir arm. Summary 96 week results from the SINGLE trial are shown in Table 2. Although the results are encouraging, there is limited data available on the use of DTG in either pregnant women or people taking rifampicin-based treatment for TB. There is also very little data available on the long-term use of DTG in combination with TDF/FTC, and no data are available on the combination with TAF/FTC.

Rifampicin is known to significantly lower plasma concentrations of DTG [27]. The current advice, based on this study, is to use a double dose of DTG (50 mg twice daily) with rifampicin, but there is very limited clinical experience with this combination, particularly in TB co-infected patients.

There is limited safety or efficacy data available on the outcomes of treating with DTG in pregnancy. There is the possibility that certain calcium or iron supplements frequently used during pregnancy could significantly reduce DTG drug levels [28]. The prescribing information for DTG contains a caution regarding the possibility of patients experiencing immune reactivation syndrome within a few weeks of starting treatment or of later developing autoimmune disorders, such as Graves’ disease [29]. Immune reconstitution syndrome may be a particular issue for people co-infected with tuberculosis. In the registrational trials programme for DTG there is very little clinical experience of first-line treatment for people with tuberculosis. This lack of supportive data introduces uncertainty about whether first-line DTG can be recommended as the first-choice treatment for large-scale access programmes until there is clinical experience of their use either in pregnancy or rifampicin-based TB treatment.

Pharmacokinetic and clinical studies of DTG in pregnancy and when co-administered with TB drugs are being planned or are in progress [30,31]. This information, together with accumulating clinical and programmatic experience on the use of DTG in countries where it is already being used, will allow for a more complete consideration of potential role of these new drugs in first- and/or second-line therapy globally.

### TAF versus TDF in first line

TAF is a prodrug of tenofovir, which is boosted significantly by either ritonavir or cobicistat. In one pharmacokinetic study, the mean intracellular concentrations of tenofovir diphosphate were 6.5 times higher using TAF compared to TDF, whereas the mean plasma tenofovir exposure was 91% lower [32].

The dose of TAF is 10 mg once daily when co-administered with ritonavir or cobicistat (to adjust for their boosting effects from the inhibition of p-glycoprotein) or 25 mg once daily when given without these booster drugs. This dosing is supported by a recently published pharmacokinetic study showing that the plasma concentration of tenofovir was bioequivalent between TAF 10 mg once daily given with cobicistat and TAF 25 mg given without cobicistat [33]. Importantly, the pharmacokinetics of TAF 25 mg once daily are food dependent: the area under the curve is up to 35% lower when given in the fasted state, compared with a moderate fat meal [34].

TDF levels are also raised significantly when co-administered with either cobicistat or three ritonavir-boosted PIs – lopinavir, darunavir (DRV) and atazanavir (ATV) [35–39]. Summary results are shown in Table 3. Results from randomized trials and cohort studies have shown an increased risk of renal adverse events when TDF is used in combination with a ritonavir-boosted PI and when tenofovir plasma concentrations are high [40–43].

**Table 3:** Effects of either cobicistat or ritonavir-boosted protease inhibitors on tenofovir pharmacokinetics [35–38]

	Effect on tenofovir		
	
Drug	C_max_	AUC	C_min_
Cobicistat	1.34 (1.34 to 1.78)	1.23 (1.16 to 1.38)	1.25 (1.16 to 1.36)
Lopinavir/r	1.15 (1.07 to 1.22)	1.32 (1.25 to 1.38)	1.51 (1.37 to 1.66)
Atazanavir/r	1.34 (1.20 to 1.51)	1.37 (1.30 to 1.45)	1.29 (1.21 to 1.36)
Darunavir/r	1.24 (1.08 to 1.42)	1.22 (1.10 to 1.35)	1.37 (1.19 to 1.57)

Geometric mean ratio (90% confidence intervals).

However, patients are routinely treated with TDF at the standard 300 mg once-daily dose in combination with either cobicistat or ritonavir, without dose adjustments, despite the known boosting effects of these pharmacoenhancers.

In the Phase 2 and 3 clinical trials of TAF versus TDF, the dose of TAF was reduced from 25 to 10 mg once daily, to compensate for the boosting effects of cobicistat or ritonavir. However, the dose of TDF was not reduced in these studies [9,32,44]. This lack of dose adjustment for TDF could therefore introduce a bias in the comparison of safety between TAF and TDF in the current randomized trials.

There have been two Phase 2 trials of TAF versus TDF conducted and two Phase 3 trials [9,32,44]. Results are shown in Table 4. Overall, there was no difference in efficacy of TAF over TDF across the four studies in terms of the percentage of patients with HIV RNA <50 copies/mL at Week 48 or 96, the number of virological failures or the risk of treatment-emergent drug resistance. There was also no significant difference in the risk of clinical adverse events between TAF and TDF. Patients receiving TAF were more likely to show increases in LDL cholesterol and total cholesterol plasma levels, as tenofovir tends to lower lipid levels. By contrast there was a statistically significant difference in some toxicity biomarkers (eGFR and bone mineral density) in favour of TAF at 48 weeks analysis. In the pivotal Phase 3 trials, the differences in lipid, bone and renal markers seen at 48 weeks did further diverge at 96 weeks [9]. It is not clear whether these differences in lipid, renal or bone markers will translate into clinically significant differences in the risk of adverse events with long-term use (e.g. myocardial infarction, proximal renal tubulopathy, Fanconi syndrome or bone fractures).

**Table 4:** Summary Week 48 or Week 96 efficacy and safety results from randomized trials of first-line TAF versus TDF

Clinical trial	TAF 10 mg+FTC	TDF 300 mg+FTC
Darunavir Phase 2 trial (Week 48 results) [32]		
Treatment arms	TAF/FTC/DRV/COBI	TDF/FTC/DRV/COBI
Sample size	103	50
HIV RNA<50 copies/mL	7%	84%
Virological failure	6%	4%
Drug resistance	0%	0%
Discontinuation for adverse events	2%	4%
Elvitegravir Phase 2 trial (Week 48 results) [44]		
Treatment arms	TAF/FTC/ELV/COBI	TDF/FTC/ELV/COBI
Sample size	112	58
HIV RNA <50 copies/mL	88%	88%
Virological failure	3%	5%
Drug resistance	0%	3%
Grade 3 or 4 clinical adverse events	10%	5%
Grade 3 or 4 laboratory adverse events	25%	17%
Elvitegravir Phase 3 trials (Week 96 results) [9]		
Treatment arms	TAF/FTC/ELV/COBI	TDF/FTC/ELV/COBI
Sample size	866	867
HIV RNA <50 copies/mL	87%	85%
Virological failure	5%	5%
Drug resistance	1.2%	0.9%
Grade 3 or 4 clinical adverse events	12%	12%
Grade 3 or 4 laboratory adverse events	28%	25%

COBI, cobicistat; DRV, darunavir; ELV, elvitegravir; FTC, emtricitabine; TAF, tenofovir alafenamide; TDF, tenofovir disproxil fumarate.

There are several problems with the clinical development programme for TAF that could limit its potential to be used in low-middle income countries (LMICs). The current clinical trial programme only includes first-line treatment of TAF 10 mg + FTC in combination with either DRV/cobocistat or elvitegravir/cobicistat – these combinations are judged to be too expensive to mass produce (Table 1] and therefore not suitable for a public health approach in low income countries [13,14].

Unit costs of treatment would be far lower if TAF and 3TC or FTC could be combined with DTG (50 mg once daily), which does not require pharmacokinetic boosting. In addition, if TAF were combined with either EFV or DTG the 25 mg once-daily dose would need to be used. There are no randomized trials to show whether the 25 mg dose of TAF will show the same efficacy and safety profile as TAF 10 mg boosted by cobicistat. There may be no clinically significant safety advantage of TAF 25 mg once daily over tenofovir 300 mg, in the absence of cobicistat or ritonavir.

There are two additional issues that need to be addressed before widespread use of TAF can be recommended for treatment. First, the activity of TAF against hepatitis B has been found to be similar to TDF in a Phase 2 study, but the drug is currently not registered for use for this indication [45]. Second, the drug interaction between TAF and rifampicin needs to be evaluated in detail, as TAF may be susceptible to induction. A final consideration is that while TDF is currently recommended for HIV pre-exposure prophylaxis, there are major trial design challenges that complicate the assessment of TAF for this indication.

### Cobicistat versus ritonavir as a pharmacokinetic booster

Co-formulated tablets containing either ATV or DRV with the pharmacokinetic booster cobicistat (150 mg once daily) [46] have recently been approved in Europe and North America. In addition, the integrase inhibitor ELV has been combined with cobicistat 150 mg once daily as part of a single pill co-formulation with TDF/FTC. In Europe and North America, co-formulated tablets of either ATV or DRV with ritonavir have not been developed. However these tablets have been recently developed as generics and a once-daily heat-stable co-formulation of ATV and ritonavir has been recently approved for use in LMICs [13,47].

### ATV/cobicistat

In a pharmacokinetic study of healthy volunteers, cobicistat 150 mg and ritonavir 100 mg once daily had bioequivalent boosting effects on the pharmacokinetics of ATV [48]. In a Phase 3 study of first-line treatment, ATV plasma Ctrough was 7 to 16% lower for ATV/cobicistat 150 mg compared with ATV/ritonavir [10,49]. A large non-inferiority trial has been conducted to compare the efficacy and safety of ATV 300 mg once daily, boosted with either 100 mg ritonavir or 150 mg cobicistat [10]. This trial showed non-inferior efficacy for the ATV/cobicistat arm versus the ATV/ritonavir arm, with similar safety profiles in the two arms.

### DRV/cobicistat

In the main pharmacokinetic study of DRV, conducted in healthy volunteers, the Cmin of DRV 800 mg once daily was significantly lower when boosted with cobicistat (mean 1478 ng/mL) compared to boosting with ritonavir (2015 ng/mL) [50]. In the discussion of this trial the authors suggested that any reduction in the DRV Cmin up to 50% should not adversely affect the efficacy profile of DRV [50]. A fully powered non-inferiority study has not been conducted to compare the efficacy of DRV/cobicistat versus DRV/ritonavir despite the significantly lower Cmin for DRV/cobicistat observed in the pharmacokinetic studies. Instead, there has been a non-randomized study evaluating the efficacy of DRV/cobicistat as a first-line treatment [51]. It is difficult to judge from this study whether the efficacy of DRV/cobicistat 800/150 mg dose is equivalent to the original DRV/r 800/100 mg dosing.

### Generic co-formulations with ritonavir

In LMICs, there are already co-formulated pills available, combining ATV and ritonavir (300/100 mg once daily) [26,47]. There are also development programmes in place to produce generic co-formulated heat-stable pills containing DRV and ritonavir, which are expected to be available by the end of 2016. Cobicistat does not have anti-HIV activity and so should not lead to the development of PI resistance. However in clinical trials of boosted PIs, the risk of treatment-emergent PI resistance is very low (Table 5]. Therefore the lack of anti-HIV activity may not actually be a significant advantage for cobicistat over ritonavir.

**Table 5:** Summary Week 48 or Week 96 efficacy and safety results from randomized trials of PI/3TC combinations versus triple therapy

Clinical trial [reference]	PI/r+1 NRTI	Triple ARV therapy
GARDEL (first-line, 96 weeks) [12]	LPV/r+3TC (*n=*217)	LPV/r+2 NRTIs (*n*=209)
HIV RNA<50 copies/mL	90%	84%
Virological failure	2.4%	2.1%
Drug resistance	*n=*4	*n=*3
Discontinuation for adverse events	0.6%	2.8%
SALT (maintenance, 96 weeks) [58]	ATV/r+3TC (*n*=143)	ATV/r+2 NRTIs (*n*=143)
HIV RNA<50 copies/mL	69%	69%
Virological failure	7.5%	5.2%
Drug resistance	*n*=0	*n*=1
Discontinuation for adverse events	5%	8%
ATLAS-M (maintenance, 48 weeks) [56]	ATV/r+3TC (*n*=133)	ATV/r+2 NRTIs (*n=*133)
Sample size	133	133
HIV RNA<50 copies/mL	90%	80%
Virological failure	*n=*2	*n=*6
Drug resistance	*n=*0	*n=*1
Discontinuation for adverse events	3%	6%
OLE (Maintenance, 48 weeks) [57]	LPV/r+3TC (*n=*118)	LPV/r+2 NRTIs (*n=*121)
HIV RNA<50 copies/mL	88%	87%
Virological failure	2%	2%
Drug resistance	*n=*1	*n=*0
Discontinuation for adverse events	*n=*1	*n=*4

3TC, lamivudine; ARV, antiretroviral; ATV/r, atazanavir/ritonavir; LPV/r, lopinavir/ritonavir; NRTI, nucleoside reverse transcriptase inhibitor; PI, protease inhibitor.

A programmatic recommendation to switch from ritonavir to cobicistat does not seem to be justified given the lack of efficacy or safety benefits in the randomized clinical trials reported.

### The potential for a switch to two-drug combinations

There could be safety and cost benefits to using two-drug combinations that do not include nucleoside analogues such as TDF or ABC. Moreover the complexity and cost of treatment could potentially be lowered by using fewer drugs. However, results from well-powered randomized trials are needed to establish non-inferior efficacy for two-drug treatments, in order to justify the changes in treatment guidelines. Clinical trials have already been conducted in naïve patients, pretreated patients with detectable HIV RNA or as a switch option for those with HIV RNA suppression on other treatments.

### Two-drug combinations of PIs and RAL

Several large randomized trials have evaluated combinations of a boosted PI with either an integrase inhibitor (mainly RAL) or a nucleoside analogue (mainly 3TC) [52,53]. In the NEAT 001 study, first-line treatment with DRV/r plus the integrase inhibitor RAL showed similar efficacy compared with DRV/r plus two nucleoside analogues (TDF/FTC) [11]. However, the efficacy of the PI-integrase inhibitor combination was lower than the control arm for patients with baseline HIV RNA above 100,000 copies/mL, as well as in those with baseline CD4 counts below 200 cells/µL [11]. In a meta-analysis of seven clinical trials in 1270 patients, the efficacy of PI + RAL treatment was 10% lower than standard triple combination treatment (*p*<0.01) [53]. Five of these seven trials were in treatment-naïve patients, whereas two were switching studies for people with HIV RNA suppression at baseline.

Two studies (EARNEST and SECOND LINE) have evaluated lopinavir/ritonavir (LPV/r) plus RAL versus LPV/r plus two nucleoside analogues for second-line treatment. Both studies showed non-inferior efficacy for the two-drug combinations, but no clear benefits in either safety or cost [54,55]. RAL is currently more expensive than two nucleoside analogues in most countries [26,47] and has the further disadvantage of requiring twice-daily dosing.

Overall, these trial results do not suggest that combinations of LPV/r or DRV/r with RAL should replace the current standard of care in either first or second-line treatment. However, it is possible that other combinations of protease and integrase inhibitors (e.g. DTG plus DRV/r) could show more promising results.

### Two-drug combinations of PIs and 3TC

Four recently completed studies compared boosted PIs in combination with a single nucleoside analogue – 3TC. Summary results are shown in Table 5. One study, GARDEL [12], was in first-line treatment, whereas three other studies, OLE, SALT and ATLAS [56–58], recruited people with undetectable HIV RNA at baseline. Overall, these studies have shown non-inferior efficacy for PI/r + 3TC combination treatment versus triple combination treatment, within the -10% non-inferiority margin normally used for regulatory approval [53]. These results are supported by the 72-week Kalead study, which showed similar efficacy for first-line LPV/r + TDF compared with LPV/r + 2 nucleoside reverse transcriptase inhibitors (NRTIs) [59]. However, these trials have been mainly conducted in people with HIV RNA suppression at baseline or treatment-naïve patients who have been tested for drug resistance. These inclusion criteria could make it hard to translate the results into routine clinical practice in LMICs, where genotyping pretreatment is not currently the standard of care and where viral load monitoring is still being introduced.

### Other two-drug combinations

The PADDLE trial is evaluating the dual combination of DTG + 3TC versus standard triple combination treatment [60]. The initial results are impressive – all 20 patients had HIV RNA suppression <50 copies/mL within eight weeks of starting treatment. However this trial has results available only to 24 weeks of treatment, enrolled people with HIV RNA below 100,000 copies/mL and also required drug resistance testing at baseline to establish that patients were fully sensitive to the two-drug combination. There are several other ongoing trials evaluating novel dual therapies that need to be assessed as results emerge.

### The need for new clinical trials

As shown in Table 1, the combination of TAF with either 3TC or FTC, and DTG could potentially be produced for $60 per person-year when production has been fully up-scaled. This would be a significant saving on the cost of TDF/3TC/EFV. In addition this new combination may have an improved safety profile and a higher barrier to the development of drug resistance. However, this combination treatment would need to be evaluated in a large non-inferiority trial, to justify a recommendation for use as first-line treatment in millions of people.

One possible design is a three-arm study, comparing 96 weeks of first-line treatment with either TAF/FTC/DTG, TDF/FTC/DTG or TDF/FTC/EFV 600 mg. Such a trial could evaluate whether the efficacy of TAF/FTC/DTG is non-inferior to either TDF/FTC/DTG (currently recommended in most international treatment guidelines) or to TDF/FTC/EFV 600 mg (which is the most widely used first-line treatment worldwide). This trial should be conducted mainly in low-income countries to ensure that the results are applicable to mass treatment programmes in these countries.

In this trial, it would also be important to evaluate outcomes in people co-infected with TB or hepatitis B, and pregnant women. Sub-studies should assess whether there are differences between arms in lipid, bone or kidney markers.

It is important that such a trial be conducted to be consistent with standard clinical practice in LMICs. Trial inclusion criteria should be as wide as possible to include the majority of treatment-naïve patients presenting in LMICs. The use of resistance testing at screening would not be appropriate, as it is not current clinical practice in LMICs. Monitoring for viral load should not be overly frequent compared with local clinical practice. Sample sizes for HIV non-inferiority trials have been well established [61] – these trials typically include at least 300 to 350 patients per arm, evaluated for at least 96 weeks on randomized treatment.

A similar methodology could be used to establish the efficacy and safety of new dual combination treatments, if results from the ongoing clinical trials programme continue to be promising. Table 6 shows the key planned and ongoing clinical trials needed to establish new treatments – EFV 400 mg, dolutegravir and tenofovir alafenamide – in future large-scale HIV treatment programmes.

**Table 6:** Key clinical trials needed for mass treatment programmes

New treatment options	Clinical trials needed	Current status [reference]
Efavirenz 400 mg once daily	Clinical experience in pregnancy	Ongoing [25]
	Clinical experience in TB treatment	Planned
Dolutegravir	pharmacokinetics in pregnancy	Ongoing [31]
	Clinical experience in TB treatment	Ongoing [30]
	Relative efficacy vs efavirenz in LMICs	Planned
TAF 25 mg/FTC/DTG	Efficacy and safety in first-line treatment	Planned

DTG, dolutegravir; FTC, emtricitabine; LMICs, low-middle income countries; TAF, tenofovir alafenamide fumarate.

## Conclusions

The use of first-line EFV 400 mg once daily or DTG is supported by improved safety in large randomized controlled trials and will be recommended by WHO in 2016 as alternative first-line options. However, there are knowledge gaps in the efficacy and safety of these new options for populations of public health relevance (notably pregnant women and those with TB co-infection), and complementary studies are urgently needed.

A new randomized trial would be needed to establish the efficacy and safety of TAF in combination with 3TC or FTC and DTG versus TDF combined with 3TC or FTC, and DTG. Efficacy and safety results from such a non-inferiority trial could then justify up-scaled production in the future if results were favourable. If this trial can be completed, there is the potential to substantially lower the costs of first-line treatment for millions of people, while also improving safety.

The switch from ritonavir to cobicistat does not appear to offer advantages in terms of either efficacy, safety or cost for mass treatment programmes in LMICs. Co-formulated versions of PIs with ritonavir are already available.

It is too early to consider recommending dual therapy approaches as part of first- or second-line therapy within a public health approach. However this field is developing rapidly, and treatment guidelines may need to be revised as new clinical trial results emerge in the next one to two years.

Despite the continuous growth in the number of people accessing antiretroviral therapy in the last decade, there is still a need to continue treatment scale-up towards Universal Access goals, which will require sustained supplies of antiretrovirals for up to 37 million people living with HIV. If the safety of antiretroviral treatment can be improved while also simplifying regimens and lowering costs, it will become more feasible to increase the uptake of treatment in the future.
